# Assessing anti-doping knowledge among Taiwanese pharmacists

**DOI:** 10.1186/s12909-023-04795-z

**Published:** 2023-10-27

**Authors:** Yun-Chen Lee, Chung-Yu Chen, Ya-Yun Cheng, Mei-Chich Hsu, Ting-Ting Chen, William Chih-Wei Chang

**Affiliations:** 1 School of Pharmacy, College of Pharmacy, Kaohsiung Medical University, Kaohsiung, 807 Taiwan; 2 School of Medicine, College of Medicine, National Sun Yat-Sen University, Kaohsiung, 804 Taiwan; 3 Department of Sports Medicine, College of Medicine, Kaohsiung Medical University, Kaohsiung, 807 Taiwan; 4 Department of Leisure Industry and Health Promotion, College of Humanities and Management, National Ilan University, Ilan County 260, Yilan, Taiwan; 5 Doctoral Degree Program in Toxicology, College of Pharmacy, Kaohsiung Medical University, Kaohsiung, 807 Taiwan

**Keywords:** Pharmacy education, Sport, Athlete, Chinese Herbal Medicine, Performance-enhancing drug, World Anti-Doping Agency

## Abstract

**Background:**

Taiwan’s unique health behaviour, such as extensive exposure to Chinese Herbal Medicine (CHM), has introduced a risk of inadvertent doping among competing athletes. Pharmacy professionals have an imperative role in advising athletes on the safe use of medicines. This study provides an overview of anti-doping knowledge and educational needs among pharmacists in Taiwan and examines influencing factors.

**Methods:**

A cross-sectional online questionnaire survey consisting of five domains, namely demographic characteristics, source of prohibited substances, identification of prohibited substances, understanding of doping control, and education needs on anti-doping, was distributed to the registered pharmacists in Taiwan. In total, 491 responses were included in the analyses.

**Results:**

Respondents (65% female, aged 41.9 ± 11.4 years, with 68% having a Bachelor’s degree) reported a moderate anti-doping knowledge score of 37.2 ± 4.9, ranging from 21 to 48 (out of 51). Fifteen per cent of them had the experience of being counselled about drug use in sports. Higher knowledge scores were observed in younger respondents, showing an age-dependent effect (*p* < 0.001). Individuals practising in southern Taiwan (compared to northern Taiwan) and those working at clinics (compared to hospitals) exhibited lower knowledge. Most of the respondents (90%) knew that stimulant ephedrine is prohibited in sports, but few had recognised diuretic furosemide (38%) and CHM (7%) containing β_2_-agonist higenamine. Approximately 90% of respondents agreed with the need for anti-doping education.

**Conclusions:**

This study highlights the heterogeneity of anti-doping knowledge among pharmacy professionals and provides practical relevance in organising future educational topics and research-based activities.

**Supplementary Information:**

The online version contains supplementary material available at 10.1186/s12909-023-04795-z.

## Introduction

The integrity of competitive sports is maintained thanks to a worldwide anti-doping programme led by the World Anti-Doping Agency (WADA). WADA annually publishes the Prohibited List [1] and implements testing strategies such as the Athlete Biological Passport [2] to combat deliberate doping, which includes the use of anabolic steroids, erythropoiesis-stimulating agents, and human growth hormone. However, many cases of Adverse Analytical Findings (AAF) where athletes argued that their acts were unintentional still resulted in sanctions, albeit some were cleared after tremendous effort. A Thai badminton player who ranked world number 1 in women's singles, committed an anti-doping rule violation (ADRV) due to the AAF of clenbuterol, a growth promoter for animals. She has sufficiently demonstrated that the source was from contaminated meat and bears no fault or negligence; no period of ineligibility has been imposed after half a year of investigation [3]. A Russian figure skater was suspended as her sample returned an AAF of trimetazidine one day after her team won a gold medal at the Beijing 2022 Winter Olympics. Her positive drug test has been blamed on drinking a contaminated glass of water that contained her grandfather’s heart medication [4]. Unfortunately, she could face a ban of up to four years. Although athletes ought to exercise a high level of caution before taking any drug, the help of healthcare professionals can undoubtedly be supportive in practice concerning doping prevention.

The Prohibited List is published by the WADA no less than once yearly. The criteria for inclusion of a substance in the List are defined in Article 4.3 of the WADA Code [5], the highest-level document. The latest List [1] is divided into three major categories: prohibited substances (S), prohibited methods (M) and substances prohibited in particular sports (P). Prohibited substances represent the main category, which is subdivided into nine subcategories. Substances in subcategories S6 − S9 are controlled in-competition only, including stimulants (e.g., ephedrine, methylphenidate, cocaine, and amfetamines), narcotics, cannabinoids (excepting cannabidiol), and glucocorticoids when administered by injectable, oral, or rectal route. The main sections of substances prohibited at all times are subcategories S1 − S5. These are anabolic agents, peptide hormones, growth factors, related substances and mimetics, β_2_-agonists (excepting four substances taken by inhalation for limited doses), hormone and metabolic modulators (e.g., clomifene and insulin), and diuretics and masking agents (e.g., furosemide and hydrochlorothiazide).

The International Pharmaceutical Federation (FIP) published guidelines – The role of the pharmacist in the fight against doping in sport [6] clarifies that pharmacists in supporting athletes can act as a gatekeeper to recognise which medicines contain a prohibited substance and to provide information about the benefits and risks of using nutritional supplements. Despite the guidelines and ongoing educational opportunities, the Anti-Doping e-Learning Platform (ADeL) course for medical professionals launched by WADA for instance, the extent of practice and knowledge of pharmacists in preventing doping is continually being explored from country to country. Recent studies involving surveys or questionnaires concerning pharmacy professionals’ knowledge towards drugs in sports encompass pharmacists from hospital and community pharmacists in Qatar (*n* = 300) [7], US chain pharmacies (*n* = 143) [8], pharmacists in Australia (*n* = 135) [9], community pharmacists in Dessie, Ethiopia (*n* = 61) [10], pharmacists in Finland (*n* = 246) [11], community pharmacists in Malaysia (*n* = 384) [12], and community pharmacists in Sydney, Australia (*n* = 100) [13]. These studies coincide most nearly with the key findings that the majority of pharmacists identified their lack of anti-doping knowledge and confidence to advise athletes.

Taiwan has a compulsory universal healthcare system with population coverage reaching 99% that has taken the top spot in the world under the Health Care Index category for 2023 [14]. As of 2022, health facilities in Taiwan, including around 420 hospitals, 12,000 clinics, and 8200 pharmacies, provide medical services with a high international reputation [15]. Notably, over 300 types of Chinese Herbal Medicine (CHM) single herb preparations and over 500 types of CHM formulas are covered by Taiwan’s healthcare [16]. According to a previous investigation, the widespread popularity of CHM in Taiwan can be manifested in more than 50% of citizens having used CHM during the 6-year studied interval [17]. This unique health behaviour could be traced back to the traditional culture thousands of years ago but recently has also introduced a significant risk of inadvertent doping to competing athletes. Athletes could be exposed to steroids found in musk, ephedrines in Ephedrae Herba and Pinelliae Rhizoma, and higenamine in Plumula Nelumbinis and Aconiti Tuber [18–21]. Pharmacy professional has therefore an imperative role in advising the safe and rational use of medicines to athletes. Japan Anti-Doping Agency initiated a unique Sports Pharmacist system in 2009 to deliver education programmes and credentialling for sports pharmacists [22]. However, there remain limited opportunities for formalised or harmonised education for pharmacy professionals in Taiwan and worldwide.

Even though the key roles of pharmacists in assisting athletes have been established, and the specific medical practices in Taiwan's healthcare system have been identified, little is known about Taiwanese pharmacists' knowledge and educational needs on anti-doping issues. One pilot study [23] targeted domestic medical personnel who joined the training courses of anti-doping in a local hospital. Among the approached 42 pharmacists, 24% (*n* = 10) had experienced athlete counselling at some point in their careers. Only 5% of them appeared to be confident in helping athletes against doping, and 62% had never heard of the WADA's list. However, the overall levels of knowledge about doping in sports and their heterogeneity among educational backgrounds, practising statuses, and/or accessibility to resources have not been well studied. This study aimed to provide insights into the variables that may affect registered pharmacists' levels of anti-doping knowledge. Identifying the areas of deficiencies can help future curriculum development by indicating which topics should be emphasised and could certain groups of pharmacists benefit most from. The findings will aid in organising research-based educational activities for sports pharmacy in Taiwan.

## Methods

### Study setting and ethics

This study is a national cross-sectional online survey that was conducted in September 2022 targeting registered pharmacists in Taiwan. The study is approved by the Institutional Review Board, Kaohsiung Medical University Chung-Ho Memorial Hospital (reference: KMUHIRB-E(I)-20,220,158).

### Questionnaire development process

The electronic survey questionnaire was developed using SurveyCake (25sprout, Taipei, Taiwan). The questionnaire design followed the seven-step process for developing a high-quality survey in medical education research presented by Artino et al. [24], including conducting a preliminary literature review, conducting interviews, synthesising the literature review and interviews, developing items, conducting expert validation, conducting cognitive interviews, and conducting pilot testing.

A scoping review was conducted, which showed that pharmacists play an important role in supporting athletes and preventing their unintentional use of prohibited substances. Since the population of interest has its geographically specific medical practice (e.g., CHM and over-the-counter), no suitable construct existed for the domestic survey after reviewing the available literature [7–13]. There is a need to develop a fit-for-purpose questionnaire. Three prospective respondents were then invited to discuss the proposed item and recommend modifications. Five main domains were identified by synthesising the literature review and interviews, including demographic characteristics, source of prohibited substances, identification of prohibited substances, understanding of doping control, and education needs on anti-doping. Following the drafting of the 1^st^ version, a panel of three medical education experts reviewed the drafted items and assessed the validity of the content. Panel members rated the questionnaire items in terms of clarity and their relevancy to the construct on a 4-point Likert scale (1 = not relevant or not clear, 2 = item needs some revision, 3 = relevant or clear but needs minor revision, 4 = very relevant or very clear) [25]. The item-level content validity index (I-CVI) was calculated and shown in Table S1. Among the original 16 items, 3 items were revised, 1 item was eliminated, and 4 items were newly added (see Text S1 for details). The demographic variables were chosen to understand whether the changes in the pharmacy education system, the different roles of practice pharmacists, or other social and cultural differences affect the survey outcome (see Table S2 for description). After modification, the 2^nd^ version of the questionnaire was piloted by three practising pharmacists and three pharmacy students. The test was triplicated over different days to check for adequate item variance and reliability. Minor revisions were made based on their feedback, particularly regarding the wording and technical functioning. The internal consistency reliability was determined with Cronbach's alpha of 0.92, indicating high reliability. The questionnaire was considered valid and reliable for use in this domestic survey.

The final questionnaire of 21 items consists of demographic characteristics (10 items), knowledge of anti-doping (10 items with 51 response options), and education needs on anti-doping (1 item with 6 response options). The assessment of knowledge in anti-doping is constructed by the summative score where a score of 1 was given for the correct answer and 0 for the wrong answer. A total score of 51 indicates 100% correct answers. The assessment of education needs on anti-doping included 5-point Likert scale questions (1 = strongly disagree to 5 = strongly agree). A higher score indicates the educational module is in greater need for the respondents. A statement at the beginning of the questionnaire mentions the response time generally within 10–15 min to complete and advises the respondents not to refer to any resources when answering the questions in the hope of reflecting the actual findings.

### Sampling and data quality control

Since the target population of this survey was the registered pharmacists in Taiwan, the minimum sample size was determined to be 379 using the Krejcie and Morgan formula which was based on the total number of 30,389 registered pharmacists by the end of 2022 (Table S2). The population was divided into four subgroups (i.e., northern, central, southern, eastern and outlying islands) based on the regions of practice, and the stratified random sampling method was employed to obtain representative samples. The invitation to registered pharmacists was posted to the groups of pharmacists’ associations in each city via the communication app. As for this online survey, the responses that came from the same IP addresses or fast responses (response time of below 200 s) were considered invalid and thus excluded. In addition, an attention check question (*Pharmacists can ensure the safe prescribing and dispensing of medication to patients*) was inserted within the survey to identify inattentive or fraudulent responses. Responses of answering strongly disagree or disagree were weeded out since supporting patient safety is the fundamental role of a pharmacist. Of the total, 491 responses were included in the analysis.

### Data analysis

Statistical analyses were performed using SPSS version 20.0. Descriptive data were presented as proportions, means, and standard deviations. Differences in anti-doping knowledge and education needs by each demographic characteristic were analysed using an independent sample t-test for two-group comparisons and one-way analysis of variance with Tukey’s posthoc for comparisons of three or more groups. The chi-square test was used to identify the differences in correct answer rates for the knowledge questions and percentages of the degree of agreement for the education topics between groups of each demographic characteristic. Simple linear regression was used to model the relationship between the knowledge score and the continuous variable (i.e., the respondent’s age). A *p*-value < 0.050 was considered statistically significant.

## Results

**Fig. 1:**
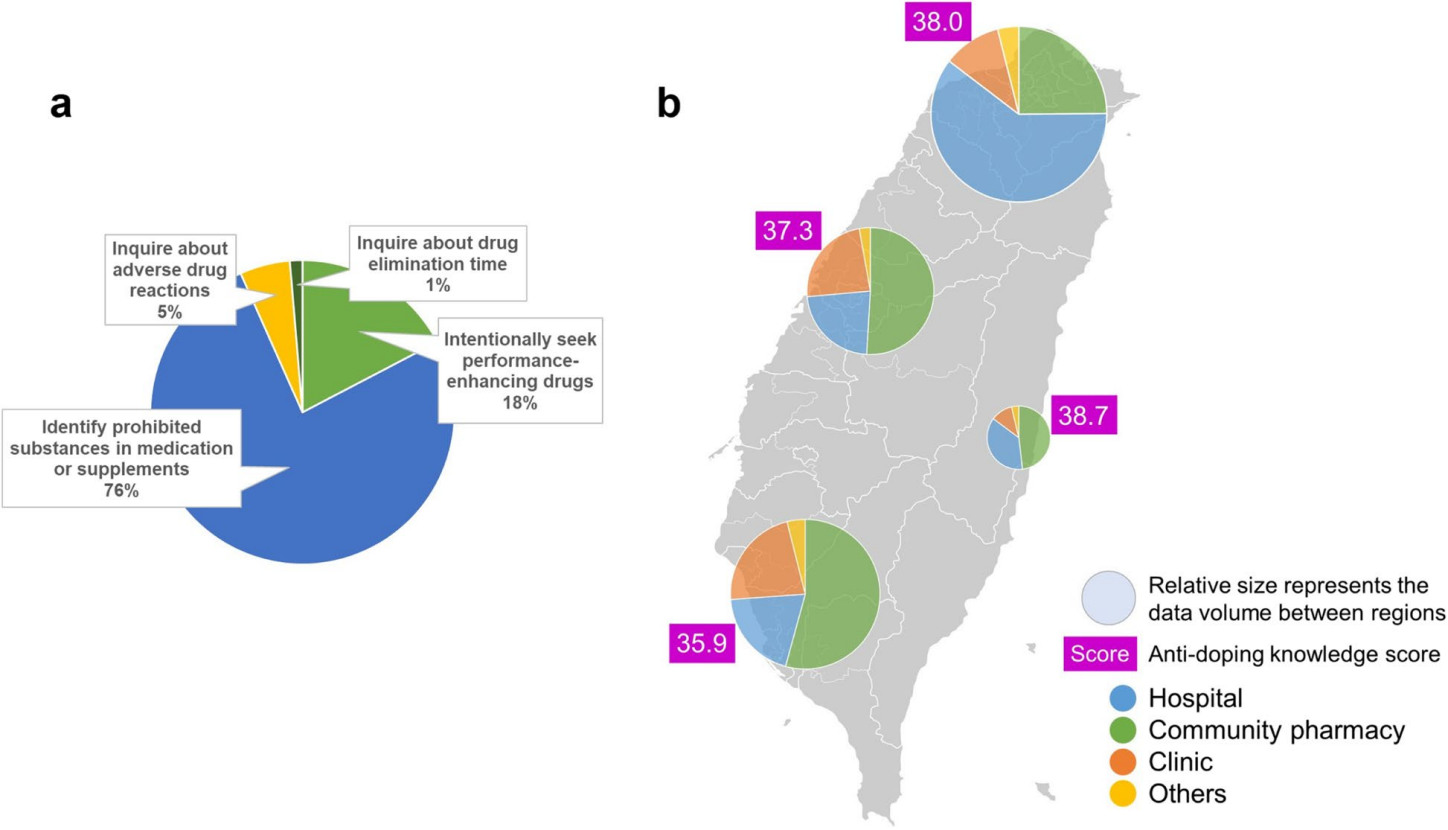
**a** Types of inquiries about drug use in sports encountered by the registered pharmacists in Taiwan (*n* = 75). **b** The map of respondents’ workplace proportion and anti-doping knowledge score distribution among northern, central, southern, and eastern Taiwan

The majority of the respondents were female (64.6%) (see Table 1). The mean age was 41.9 ± 11.4 years, ranging from 23 to 74 years. Most of the respondents obtained a Bachelor's degree (68.4%) rather than a postgraduate degree (19.3%) or an associate degree (12.2%). The primary workplace of the respondents was community pharmacy or drugstore (40.9%), hospital (38.3%), and clinic (17.1%). Notably, 15.3% of the responding pharmacists (*n* = 75) had the experience of being counselled about drug use in sports during their practice. Among these experiences encountering athletes or supporting personnel (Fig. 1a), 76.0% inquired about identifying prohibited substances in medication or supplements and 17.3% intentionally sought performance-enhancing drugs.

**Table 1:** The respondents’ demographic characteristics (*n* = 491)

Demographic variables	Number	Percentage (%)
**Gender**		
Male	174	35.4
Female	317	64.6
**Age**		
20–29 years	79	16.1
30–39 years	147	29.9
40–49 years	140	28.5
> 50 years	125	25.5
**Academic qualification**		
Associate degree	60	12.2
Bachelor's degree	334	68.0
Pharm.D	2	0.4
Master's degree	91	18.5
Doctorate degree	4	0.8
**Primary workplace**		
Community pharmacy or drugstore	201	40.9
**[Type of community pharmacy or drugstore]**		
Independent	145	72.1
Chain	54	26.9
Non-NHI contract	2	1.0
Hospital	188	38.3
**[Type of hospital]**		
Medical centre	76	40.4
Regional	69	36.7
District	43	22.9
Clinic	84	17.1
Government	6	1.2
Pharmaceutical industry or wholesale	11	2.3
Unemployed	1	0.2
**Region of practice**		
Northern Taiwan	205	41.8
Central Taiwan	106	21.6
Southern Taiwan	153	31.2
Eastern Taiwan and outlying islands	27	5.5
**Current position**		
Supervisor	87	17.7
Clinical pharmacist	27	5.5
Ambulatory care pharmacist	155	32.6
Community pharmacist	133	27.1
Pharmacist at primary care clinics	75	15.3
Others	14	2.9
**Years of practice**		
< 5 years	92	18.7
6–10 years	107	21.8
11–15 years	82	16.7
16–19 years	50	10.2
> 20 years	160	32.6
**In your practice, have you ever been counselled about drug use in sports by athletes or supporting personnel (e.g., parents, coaches, or other medical staff)? Please exemplify**		
Yes	75	15.3
No	416	84.7

The mean score of respondents’ knowledge in anti-doping was 37.2 ± 4.9 (out of a total score of 51), ranging from 21 to 48. The map of respondents’ workplace proportion and score distribution among four regions of Taiwan was displayed in Fig. 1b, suggesting the geographical heterogeneity of anti-doping knowledge. The responses from eastern Taiwan presented the highest score of 38.7 ± 3.8 whereas southern Taiwan had the lowest score of 35.9 ± 5.3. The greater composition of the responses in central, southern, and eastern Taiwan was from community pharmacies (around 50%). The responses in northern Taiwan instead came mostly from hospitals (61%), as did eastern Taiwan, which had a considerable number of responses (37%) from hospitals. Furthermore, the rates of athlete counselling experience remarkably differed between workplaces, in descending order, community pharmacies or drugstores (21.4%), hospitals (11.2%), and clinics (9.5%).

The results of correct answer rates for each knowledge question in three domains are given in Table 2. Of the ‘source of prohibited substances’ domain, the overall respondents ingrained that Western medicine could contain prohibited substances but somewhat showed moderate to poor perception that CHM (64.6%), supplement (42.2%), and even food (23.8%) could do so. Interestingly, the respondents believed that pharmacist (66.8%) is less likely than physicians (75.8%) to potentially provide prohibited substances. Of the ‘identification of prohibited substances’ domain, 90% of the respondents very much knew that pseudoephedrine (a stimulant) is prohibited in sports and ibuprofen (a non-steroidal anti-inflammatory drug) use is fine for athletes. The respondents had acceptable knowledge of triamcinolone acetonide (a glucocorticoid, 70.7%) being included on the List, yet poor understanding that furosemide (a diuretic, 38.3%) is prohibited at all times. Regarding over-the-counter (OTC) in this survey, their ingredients and indications are provided in Table S4. The respondents could sense that Suzulex Bien A Capsule, produced by a domestic pharmaceutical company U.C. Pharma, contains ephedrine (76.6%), however, showed not quite familiar with the actual ingredients of the products marketed by foreign pharmaceutical companies, Pabron "Taisho" by Taisho Pharmaceutical (Japan) containing ephedrine (55.0%) and Otrivin Moisturizing Nasal Metered-Dose Spray 0.05% by GlaxoSmithKline (UK) not containing ephedrine (55.6%). Regarding CHM, the respondents, likewise, precisely identified Ephedra Herb as containing multiple ephedrines (91.4%). On the contrary, very few had recognised Clove (17.9%) and Lotus Seed (6.5%) containing the β_2_-agonist higenamine. Regarding the common supplements supporting workout and joint mobility, the respondents fairly knew whey protein (88.2%), branched-chain amino acid (82.7%), and undenatured collagen type II (86.4%) are not banned. Nevertheless, only half of the respondents correctly identified creatine (57.2%) as unprohibited and diosgenin-containing dehydroepiandrosterone (45.6%) as prohibited. Of the ‘understanding of doping control’ domain, the criteria for including a substance on the List of ‘represent a health risk to the athlete’ (63.7%) was less known than ‘enhance sports performance’ (88.2%) among the respondents. As for athletes’ testing, more respondents knew about the collected samples of urine and blood for testing (89.4%) than the testing period of any time (55.2%). Moderate correct answer rates, ranging from 60.1% to 83.7%, were observed for the situational questions concerning athletes’ principle of strict liability during doping control.

**Table 2:** The respondents’ knowledge in anti-doping (*n* = 491)

Domain	Variables	Correct answer	Respondents with correct answer, n (%)	Respondents with wrong answer, n (%)
**Source of prohibited substances**	**Could any of the followings contain prohibited substances?**			
	Prescription medicine	True	431 (87.8)	60 (12.2)
	Over-the-counter medicine	True	373 (76.0)	118 (24.0)
	Chinese herbal medicine	True	317 (64.6)	174 (35.4)
	Supplement	True	207 (42.2)	284 (57.8)
	Food	True	115 (23.8)	374 (76.2)
	**Who can potentially provide prohibited substances?**			
	Physician	True	372 (75.8)	119 (24.2)
	Pharmacist	True	328 (66.8)	163 (33.2)
	Coach	True	385 (78.4)	106 (21.6)
	Athletic trainer	True	301 (61.3)	190 (38.7)
	Family and friends	True	383 (78.0)	108 (22.0)
	Internet	True	375 (76.4)	116 (23.6)
**Identification of prohibited substances**	**Which of the following drugs are prohibited in sports?**			
	Furosemide	True	188 (38.3)	303 (61.7)
	Chlorpheniramine	False	425 (86.6)	66 (13.4)
	Pseudoephedrine	True	442 (90.0)	49 (10.0)
	Triamcinolone acetonide	True	347 (70.7)	144 (29.3)
	Ibuprofen	False	445 (90.6)	46 (9.4)
	**Which of the following OTC products contain prohibited substances?**			
	大正百保能感冒藥 Pabron "Taisho"	True	270 (55.0)	221 (45.0)
	斯斯鼻炎膠囊 Suzulex Bien A Capsule	True	376 (76.6)	115 (23.4)
	肌立酸痛貼布 Panadol Diclofenac Oil Plaster	False	442 (90.6)	46 (9.4)
	歐治鼻去鼻塞噴劑 Otrivin Moisturizing Nasal Metered-Dose Spray 0.05%	False	273 (55.6)	218 (44.4)
	普拿疼加強錠 Panadol Extra with Optizorb	False	394 (80.2)	97 (19.8)
	**Which of the following CHM products contain prohibited substances?**			
	當歸Chinese Angelica Root	False	432 (88.0)	59 (12.0)
	麻黃Ephedra Herb	True	449 (91.4)	42 (8.6)
	丁香Clove	True	88 (17.9)	403 (82.1)
	杜仲Eucommia Bark	False	431 (87.8)	60 (12.2)
	蓮子心Lotus Seed	True	32 (6.5)	459 (93.5)
	**Which of the following supplements contain prohibited substances?**			
	Creatine	False	281 (57.2)	210 (42.8)
	Diosgenin (containing DHEA)	True	224 (45.6)	267 (54.4)
	Whey protein	False	430 (88.2)	58 (11.8)
	BCAA	False	406 (82.7)	85 (17.3)
	Undenatured collagen type II (UC-II)	False	424 (86.4)	67 (13.6)
**Understanding of doping control**	**What are the criteria for including substances on the prohibited list?**			
	Enhance sports performance	True	433 (88.2)	58 (11.8)
	Increase appetite	False	439 (89.4)	52 (10.6)
	Improve sleep	False	459 (93.5)	32 (6.5)
	Violate the spirit of sport	True	387 (78.8)	104 (21.2)
	Represent a health risk to the athlete	True	313 (63.7)	178 (36.3)
	**Which of the following statements regarding anti-doping testing are true?**			
	Testing only in-competition	False	304 (61.9)	187 (38.1)
	Testing anytime and anywhere	True	271 (55.2)	220 (44.8)
	Refusal to testing for any reason is acceptable	False	463 (94.3)	28 (5.7)
	Blood and/or urine sample for testing	True	439 (89.4)	52 (10.6)
	Skin test	False	371 (75.6)	120 (24.4)
	**Which of the following practices are to prevent inadvertent doping when using supplements?**			
	Choose supplements from a reputable and GMP company	True	371 (75.6)	120 (24.4)
	Check ingredients against the List	True	440 (89.6)	51 (10.4)
	Prioritise products that are advertised as "muscle building"	False	446 (90.8)	45 (9.2)
	Do not buy or accept supplements of unknown sources from coaches or teammates	True	442 (90.6)	46 (9.5)
	Shop online for the supplements in best-sellers	False	469 (95.5)	22 (4.5)
	**What are the consequences of an athlete taking a cold medicine prescribed by a doctor and returning a positive testing result?**			
	Athletes may commit an anti-doping rule violation	True	374 (76.2)	117 (23.8)
	Athlete may face disqualification of results, sanction, and ban	True	371 (75.6)	120 (24.4)
	Not considered a violation as athlete uses drugs unintentionally	False	404 (82.3)	87 (17.7)
	Not considered a violation as athlete uses medicines prescribed by a doctor	False	411 (83.7)	80 (16.3)
	Athlete is responsible for any banned substance found in his/her system	True	295 (60.1)	196 (39.9)

**Fig. 2:**
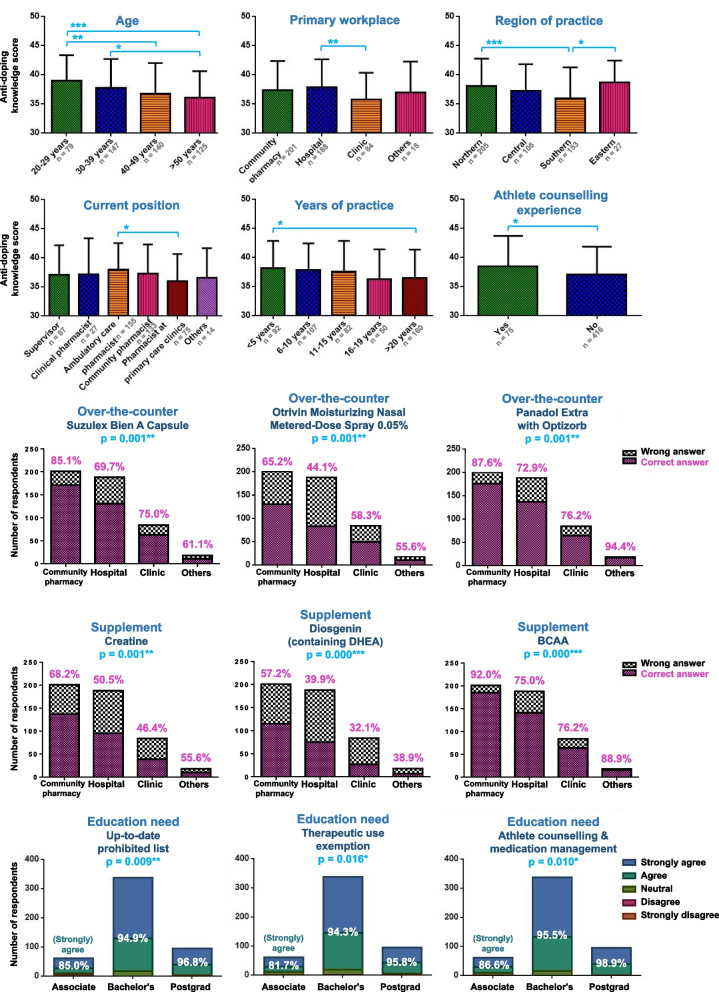
Significant statistical heterogeneity in **a** total anti-doping knowledge score within each demographic characteristic of the respondents, analysed using an independent sample t-test or a one-way analysis of variance with Tukey’s posthoc, **b** correct answer rate of each knowledge question between workplaces of the respondents, analysed using a chi-square test, and **c** degree of agreement of each education topic between academic qualifications of the respondents, analysed using a chi-square test

Significant statistical heterogeneity of the total knowledge scores was found in age, primary workplace, region of practice, current position, years of practice, and athlete counselling experience (Table 3). As shown in Fig. 2a, an age-dependent response was noted (*p* < 0.001 analysed by simple linear regression). Younger respondents had higher scores which descended by age as well as practice years. The scores of southern Taiwan were significantly lower than those of northern Taiwan (*p* < 0.001) and eastern Taiwan (*p* < 0.050). The scores of respondents working at clinics were significantly lower than that of those working in hospitals (*p* < 0.010). The respondents who had athlete counselling experience had a higher knowledge than those who did not (*p* < 0.050).

**Table 3:** Statistical significance (*p*-value) of anti-doping knowledge and educational needs among respondents’ demographic variables

Parameter	Gender ^a^	Age ^b^	Academic qualification ^b^	Primary workplace ^b^	Region of practice ^b^	Current position ^b^	Years of practice ^b^	Athlete counselling experience ^b^
Knowledge in anti-doping	0.150	**0.000**	0.098	**0.005**	**0.000**	**0.043**	**0.046**	**0.017**
Education need	0.740	0.393	0.105	0.707	0.762	0.806	0.998	0.715

^a^ Independent sample t-test

^b^ One-way analysis of variance

*p*-value in bold font denotes statistical significance (*p* < 0.050)

The mean summative score of respondents’ education needs was 26.9 ± 3.6 (out of a total score of 30). No statistical difference in the total score of educational needs was observed among respondents’ demographic variables (Table 3). While comparing six anti-doping topics, the mean score was 4.49 ± 0.67 (5-point Likert scale) suggesting the respondents’ general agreement with the need for education. However, they somehow showed less favour with the topic ‘anti-doping testing procedure’ (4.39 ± 0.72) than the others (*p* < 0.010) (Tables S5).

Figure 2b and c depicted the results with significant differences from the chi-square analyses. Of the knowledge questions, the respondents who worked in a community pharmacy had overall higher correct answer rates than the other groups in identifying prohibited substances of the OTC products and nutritional supplements. Of the education topics, the respondents who hold a higher academic qualification exhibited a greater degree of agreement in receiving anti-doping education of ‘up-to-date prohibited list’, ‘therapeutic use exemption’, and ‘athlete counselling & medication management’.

## Discussion

In the present study, we found that 15.3% of the pharmacists (75 out of 491) had been counselled about drug use in sports. This rate is far less than a study involving 246 Finnish pharmacists, where 67.9% of pharmacists reported encountering doping user groups [11]. Comparing health workforce statistics between the two countries, the numbers of medical doctors and pharmacists were 31.7 and 13.1 (/10,000 population) for Taiwan and 46.4 and 19.2 (/10,000 population) for Finland, respectively [26]. The opportunities for athletes to approach pharmacists for counselling should be similar. Of anti-doping testing statistics by national anti-doping organisations in the last three years, the AAF rates of the tested samples were 0.5% − 0.9% reported by the Chinese Taipei Anti-Doping Agency and 0.3% − 0.4% by the Finnish Center for Integrity in Sports, respectively [27]. Regardless of the effectiveness of the implemented testing programme, doping prevalence in professional athletes (e.g., international or national level) seemed not to be higher in Finland. Consequently, the remarkable difference in pharmacists’ experiences between the two studies can be attributed to other more complicated factors, for instance, the dissimilarities in the roles of pharmacists working as frontline customer service or patient counselling, or the habits of drug use among recreational-level athletes who participate in local or school competitions where testing rarely being implemented.

Pharmacists from northern and eastern Taiwan exhibited better knowledge scores than those from southern Taiwan. Part of the reason for such geographical heterogeneity could be the uneven sample distribution among the respondents’ workplaces. The northern and eastern samples comprised a more significant percentage of the responses from those who worked in hospitals (Fig. 1b), of which they obtained the highest score among all workplaces (Fig. 2a). Pharmacists who worked in community pharmacies or drugstores, despite slightly lower scores compared with hospitals, reported the highest likelihood of encountering athlete counselling. Therefore, it is suggested that future anti-doping education should have priority over community pharmacy professionals.

Pharmacists aged 20 − 29 or who practised less than 5 years obtained the highest knowledge scores, yet the scores declined along with the increase in age or practice years. To date, no available course has been incorporated into the pharmacy curricula of university education in response to FIP guidelines [6, 28]. This interesting phenomenon could be explained in part by the implementation of the postgraduate year training program since 2007, which aims to ensure that trainee pharmacists acquire proficient medication knowledge [29]. Young pharmacists are equipped with evidence-based skills and are used to acquiring information through a more versatile approach, which may, in turn, be likely to expose them to anti-doping topics. Taiwanese people have had increasing educational resources in recent years. First, anti-doping lectures become mandatory in national sports federations’ seminars for coaches and officials, as well as in high school physical education classes. Second, the Taiwan Anti-Doping Association (with the Sports Administration’s funding) launched a mobile application in 2017 allowing anyone to check over 40,000 pharmaceuticals containing a prohibited substance and an anti-doping education platform by 2021 covering at least five essential topics that youth athletes should know [30]. The above developments, among very few that have a traditional Chinese interface, are tailor-made for the domestic population. Thirdly, numerous news, blog posts, virtual seminars, and some of these targeting the audience of medical personnel, have raised awareness of the issues related to clean sport.

We noted that Ephedra and ephedrines are generally recognised by pharmacists (correct answer rate of 90%); conversely, diuretics, and CHMs containing higenamine were poorly identified. This is because the mechanism of action of diuretics is not directly linked to the enhancement of sports performance; rather, the rationale for the prohibition of diuretics is to mask the presence of other doping agents or to induce weight loss in sports with weight criteria. On the other hand, higenamine acting as a β_2_ agonist has been included in the List only since 2017. Unlike longstanding ephedrines, pharmacists are not yet familiar with this plant-origin substance. Higenamine is found in many folk medicines, including CHMs such as Aconite, Asarum Root, Clove, and Lotus Seed [20, 21]. We also found that the ability to identify certain products differs between the workplaces of practice. Community pharmacists showed notably better familiarity with OTC medications and nutritional supplements than other workplaces (Fig. 2b). Hospital pharmacists, albeit expected to have good clinical knowledge and were found to have superior overall anti-doping knowledge, present unaware of products with which they do not often come in contact.

Our results on education needs for anti-doping, around 90% responding as agree on each topic, go in line with existing literature by Lemettilä et al.[11] (pharmacists in Finland), Mottram et al. [7] (pharmacists in Qatar) and Shibata et al. [31] (pharmacy students in Japan). These studies indicated that approximately 70 − 90% of respondents expressed an interest or need for further education. Even though pharmacists reported their considerable need to receive training, the study found that only 20 − 60% expressed willingness to participate in anti-doping activities, particularly ‘doping testing’ of the poorest willingness [11]. This is in accordance with our findings that pharmacists in Taiwan were less in favour of the educational topic ‘anti-doping testing procedure’. The comparisons of pharmacist participants, instruments, and key findings between studies from other regions are summarised in Table S6.

## Limitations

Firstly, despite the mention of an adequate sample size and the use of a stratified random sampling method to ensure sample representativeness, this study acknowledges the potential for response bias. Participants were invited to complete the survey through advertisements on communication software such as LINE and Facebook. This approach makes it impossible to track the frequency of advertisements being posted or forwarded and the actual number of pharmacists reached. Therefore, calculating a response rate is not applicable. Under some circumstances, it is speculated that the observed regional differences in results may be attributed to variations in the distribution of practice settings among responding pharmacists.

Secondly, ensuring the quality of responses poses a challenge for online self-administered questionnaires. We attempted to address this issue by screening for possible invalid data, identifying fast responses, and incorporating an attention check question. Despite providing explanatory statements and explicitly instructing participants to respond honestly to questions based on their own knowledge without referring to any external resources, it remains possible that some respondents may not have adhered to these guidelines.

## Conclusions and implications

Pharmacists are perceived as specialists who provide pharmaceutical care and have, at some point, become key players as athlete support personnel in preventing doping. While previous works focused on providing an overview of anti-doping knowledge of pharmacists from a single workplace (e.g., community), the present study identifies gaps and suggests several domains where particular sub-groups of pharmacists showed lower understanding. For instance, pharmacists who are older, practice in southern Taiwan, or work at clinics exhibited these gaps. Drugs that are irrelevant to performance enhancement, such as diuretics and masking agents, as well as newly banned emerging substances found in dietary supplements or folk medicines, are less recognised by pharmacists. Additionally, it is plausible that Taiwanese pharmacists are less likely to have contact with athletes or be counselled by athletes compared to pharmacists in other countries.

This study suggests that one of the key considerations in planning future education for pharmacy professionals is selecting materials and topics that are directly relevant to their practice. For community pharmacists, it is advisable to enhance their awareness of identifying the presence of common ingredients, such as stimulant methylhexanamine, β_2_-agonist higenamine, cannabinoids, opium, and even ecdysterone in OTC products, supplements, and herbal medicines. This knowledge can also be applied to other countries that have their own traditional medicines, especially in the Asia–Pacific region, such as Japan, Korea, Malaysia, and China. For pharmacists working in medical centres, despite their generally strong clinical knowledge, the implementation of an alert system through labelling athlete identity and prohibited drugs would be very beneficial. This is particularly important for pharmacists working in places that supply thousands of medicines. Applicable tools, such as Globo DRO or relevant databases in addition to the List, should also be introduced.

As the field of sports pharmacy gains momentum and global recognition, investigating the extent of education related to drug use in sports and doping control for pharmacists' continuing professional development represents a promising avenue for future research. Subsequent studies could assess different educational aspects, including pharmaceutical care for athletes, sample collection and testing, therapeutic use exemptions, and results management, to determine which components are relevant and interesting for pharmacists.

### Supplementary Information

**Additional file 1.**

## Data Availability

The datasets generated and/or analysed during the current study are not publicly available due to proprietary nature but are available from the corresponding author on reasonable request.
