# Supplementary material for: Assessing anti-doping knowledge among Taiwanese pharmacists
Source: BMC Med Educ. 2023 Oct 27;23:808. doi: 10.1186/s12909-023-04795-z (PMC10605328; doi:10.1186/s12909-023-04795-z)
Supplement: Supplementary file 1 — Additional file 1. [file 12909_2023_4795_MOESM1_ESM.docx]

**Text S1. Revision following the expert validation**

1. Item 8 (*What is the total number of prescriptions in your institution in 2021?*) was eliminated because two experts considered it as irrelevant to the construct of this study.
2. Item 12 (*Which of the following drugs are prohibited in sports? Acetaminophen; Amlodipine; Chlorpheniramine; Clenbuterol; Clomiphene; Erythropoietin; Furosemide; Ibuprofen; Liraglutide; Omeprazole; Prednisolone; Propranolol*) was revised as (*Which of the following drugs are prohibited in sports? Furosemide; Chlorpheniramine; Pseudoephedrine; Triamcinolone acetonide; Ibuprofen*).
3. Item 16 (*How well do you know about doping control? Please use a 5-point Likert scale to answer the following questions: I understand that doping control is based on the principle of strict liability; I understand the inclusion criteria for substances prohibited in sport; I do not understand the penalties applied to athletes who violate the anti-doping rules; I understand the sample collection process of doping control (e.g., urine, blood) and the rights and responsibilities of the tested athletes; I understand the purpose of the Therapeutic Use Exemption; I do not understand the whereabouts information that athletes of the Registered Testing Pool need to provide*) was revised and divided to 5 items (Item 16–Item 20):
   - Item 16 (*What are the criteria for including substances on the prohibited list? Enhance sports performance; Increase appetite; Improve sleep; Violate the spirit of sport; Represent a health risk to the athlete*)
   - Item 17 (*Which of the following statements regarding anti-doping testing are true? Testing only in-competition; Testing anytime and anywhere; Refusal to testing for any reason is acceptable; Blood and/or urine sample for testing; Skin test*)
   - Item 18 (*Which of the following practices are to prevent inadvertent doping when using supplements? Choose supplements from a reputable and GMP company; Check ingredients against the List; Prioritise products that are advertised as "muscle building"; Do not buy or accept supplements of unknown sources from coaches or teammates; Shop online for the supplements in best-sellers*)
   - Item 19 (*What are the consequences of an athlete taking a cold medicine prescribed by a doctor and returning a positive testing result? Athletes may commit an anti-doping rule violation; Athlete may face disqualification of results, sanction, and ban; Not consider a violation as athlete uses drug unintentionally; Not consider a violation as athlete uses medicine prescribed by a doctor; Athlete is responsible for any banned substance found in his/her system*)
   - Item 20 (*What are the consequences of an athlete taking a cold medicine prescribed by a doctor and returning a positive testing result? Athletes may commit an anti-doping rule violation; Athlete may face disqualification of results, sanction, and ban; Not consider a violation as athlete uses drug unintentionally; Not consider a violation as athlete uses medicine prescribed by a doctor; Athlete is responsible for any banned substance found in his/her system*).

| **Table S1. Calculation of item-level content validity index (I-CVI)** | | | | |
| --- | --- | --- | --- | --- |
| Item | Relevant  (rating 3 or 4) | Not relevant  (rating 1 or 2) | I-CVI* | Interpretation |
| 1 | 3 | 0 | 1 | Appropriate |
| 2 | 3 | 0 | 1 | Appropriate |
| 3 | 3 | 0 | 1 | Appropriate |
| 4 | 3 | 0 | 1 | Appropriate |
| 5 | 3 | 0 | 1 | Appropriate |
| 6 | 3 | 0 | 1 | Appropriate |
| 7 | 3 | 0 | 1 | Appropriate |
| 8 | 1 | 2 | 0.33 | Eliminated |
| 9 | 3 | 0 | 1 | Appropriate |
| 10 | 3 | 0 | 1 | Appropriate |
| 11 | 3 | 0 | 1 | Appropriate |
| 12 | 2 | 1 | 0.67 | Need for revision |
| 13 | 3 | 0 | 1 | Appropriate |
| 14 | 3 | 0 | 1 | Appropriate |
| 15 | 3 | 0 | 1 | Appropriate |
| 16 | 2 | 1 | 0.67 | Need for revision |
| 17 | 3 | 0 | 1 | Appropriate |
| Note: 1 = not relevant or not clear, 2 = item needs some revision, 3 = relevant or clear but needs minor revision, 4 = very relevant or very clear | | | | |

| **Table S2. Choice of demographic variables** | | |
| --- | --- | --- |
| Demographic variable | Purpose | Description |
| Age, academic qualification, and year of practice | To understand whether the changes in pharmacy education system affect the survey outcome | The system of domestic pharmacy education has changed in the past few decades. Pharmacist licences are no longer issued to the graduates of five-year junior colleges (Associate degree) since 1980. Four- or five-year undergraduate studies in universities (Bachelor's degree) have become the mainstream for pharmacy education. The 2-year postgraduate year training program for the registered pharmacists has been implemented since 2007. The new 6-year Pharm.D. programme has been added to substantially enhance clinical pharmacy training in several universities since 2009. In 2023, 20% of the pharmacy graduates are awarded the Pharm.D. degree. |
| Workplace, job position, and practical counselling experience | To understand whether the different roles of practice pharmacists affect the survey outcome | Practice pharmacists carry out different functions, such as the sale and dispensation of pharmaceuticals, and patient care, which involves reviewing and managing patients' drug therapy. The pharmacists' ability and opportunity to perform these functions depend on their job roles. |
| Gender and region of practice | To understand whether other social and cultural differences affect the survey outcome | Some factors are related to uneven distribution of political, financial, medical, technological, and educational resources. |

| **Table S3. Number of registered pharmacists in Taiwan between regions (data of 2022)** | | |
| --- | --- | --- |
| Region and division | Distribution of 30389 registered pharmacists | Proportional distribution for estimating 500 responses |
| **Northern Taiwan** | **13628** | **220** |
| Keelung | 356 | 5 |
| Taipei | 5268 | 86 |
| New Taipei | 4238 | 69 |
| Taoyuan | 2354 | 38 |
| Hsinchu | 1034 | 16 |
| Miaoli | 378 | 6 |
| **Central Taiwan** | **6698** | **108** |
| Taichung | 4117 | 67 |
| Changhua | 1405 | 23 |
| Nantou | 484 | 7 |
| Yunlin | 692 | 11 |
| **Southern Taiwan** | **8890** | **144** |
| Chiayi | 1087 | 17 |
| Tainan | 2777 | 45 |
| Kaohsiung | 4098 | 67 |
| Pingtung | 928 | 15 |
| **Eastern Taiwan and outlying Islands** | **1173** | **19** |
| Yilan | 474 | 7 |
| Hualien | 391 | 6 |
| Taitung | 200 | 3 |
| Penghu | 57 | 1 |
| Kinmen | 40 | 1 |
| Matsu | 11 | 1 |

| **Table S4. Information of the OTC products cited in this survey** | | | | | |
| --- | --- | --- | --- | --- | --- |
| **Product** | 大正百保能感冒顆粒  Pabron granules "TAISHO" | 斯斯鼻炎膠囊  Suzulex Bien A Capsule | 普拿疼肌立  酸痛藥布  Panadol Diclofenac Oil Plaster | 歐治鼻 噴鼻液 0.1%  Otrivin Moisturizing nasal Metered-Dose Spray 0.1% | [普拿疼止痛加強錠](https://yaowu8.cn/drug/13392/)  Panadol Extra with Optizorb |
| **Ingredient** | - Acetaminophen - Caffeine anhydrous - Carbinoxamine maleate - Potassium guaiacol sulfonate - dl-Methylephedrine HCl - *Dextromethorphan HBr* | - Belladonna total alkaloids - Chlorpheniramine maleate - Caffeine anhydrous - Glycyrrhizinate Momoammonium - *Pseudoephedrine HCl* | - Diclofenac sodium | - Xylometazoline HCl | - Acetaminophen - Caffeine |
| **Indication** | Relieve various symptoms of colds (fever, headache, runny nose, nasal congestion, sneezing, coughing, phlegm) | Stuffy nose, runny nose, sneezing | Relieve local pain caused by inflammation | Stuffy nose, runny nose, sneezing | Reduce fever and relieve pain |
| Note: The underlined ingredients are substances prohibited in sports. | | | | | |

| **Table S5. The respondents’ education needs in anti-doping (n = 491)** | | | | | | | |
| --- | --- | --- | --- | --- | --- | --- | --- |
| **Question: To what extent do you agree or disagree with your need for anti-doping education on each topic?** | | | | | | | |
| **Answer: 5-point Likert scale (1 = strongly disagree to 5 = strongly agree)** | | | | | | | |
| **Topic** | **Number of responses, n (%)** | | | | | **Score (n = 491)** | |
|  | **1** | **2** | **3** | **4** | **5** | **Mean** | **SD** |
| **Up-to-date prohibited list** | 3 (0.6) | 0 (0) | 26 (5.3) | 163 (33.2) | 299 (60.9) | 4.54** | 0.66 |
| **Therapeutic use exemption** | 2 (0.4) | 1 (0.2) | 31 (6.3) | 183 (37.3) | 274 (55.8) | 4.48 | 0.66 |
| **Anti-doping testing procedure** | 2 (0.4) | 2 (0.4) | 50 (10.2) | 185 (37.7) | 252 (51.3) | 4.39 | 0.72 |
| **Athlete counselling and medication management** | 2 (0.4) | 0 (0) | 22 (4.5) | 175 (35.6) | 292 (59.5) | 4.54** | 0.62 |
| **Food and supplements with prohibited substances** | 2 (0.4) | 1 (0.2) | 20 (4.1) | 175 (35.6) | 293 (59.7) | 4.54** | 0.63 |
| **Principle of strict liability and anti-doping rules** | 2 (0.4) | 3 (0.6) | 41 (8.4) | 181 (37.1) | 263 (53.6) | 4.43 | 0.71 |
| **Significantly higher as compared with the score of ‘Anti-doping testing procedure’ (p < 0.010) | | | | | | | |

| **Table S6. The studies from other regions assessing anti-doping knowledge among pharmacists.** | | | |
| --- | --- | --- | --- |
| **Participant** | **Instrument** | **Key findings** | **Reference** |
| Qatar  n = 300 | Survey (18 questions) for knowledge, perceived role of healthcare professionals, and attitudes toward educational needs for drug use in sports | Doping and anti-doping of knowledge were low and suggesting they needed further professional training. | Mottram, D., et al. (2016) Journal of Sports Medicine and Physical Fitness. 56(6):817-24. |
| USA  n = 143 | Survey (21 questions) for providing sports supplements: (1) counseling of knowledge, confidence, and enthusiasm; (2) perceptions of counseling benefits, and barriers to implementation | 92% of pharmacists are beneficial to provide counseling on sports supplements. Perceived barriers included lack of knowledge, evidence, and time | Howard, M.S., et al. (2018) Journal of the American Pharmacists Association. 58(4):S30-S6. e2. |
| Australia  n = 135 | Survey (7 questions) for familiarity with WADA guidelines, knowledge on prohibited drug classes, and their opinion about the role of pharmacists in educating athletes on medication use | Majority indicating that they were not confident and enough knowledge to advise athletes. | Yee, K.C., et al. (2020) Pharmacy. 8(1):10. |
| Ethiopia  n = 61 | Survey (23 questions) for knowledge of topics such as doping and medical exemptions determine whether it is a drug on the prohibited list | The pharmacists having low knowledge score revealed that they need further specialized training on doping and anti-doping. | Gebregergs, H., et al. (2021) Integrated Pharmacy Research & Practice. 10:43-50. |
| Finland  n = 246 | Survey (26 questions) for pharmacists’ perceptions about doping, knowledge, and need for education about the pharmacology of doping agents, anti-doping counseling, and information source | Pharmacists self-assessed knowledge on anti-doping counselling to be poor or rather poor. Their highest needs for education were related to nutritional supplements’ doping risks, substances listed as doping agents etc. | Lemettilä, M., et al.‚ (2021) Journal of Performance Enhancement & Health. 9(2):100195. |
| Malaysia  n = 384 | Survey (23 questions) for community pharmacists’ knowledge of tackling the issue of inadvertent doping | Community pharmacists in Malaysia have limited knowledge in the field of doping. More programmes and activities related to doping and drugs in sports should be held. | Voravuth, N., et al. (2022) PLoS One. 17(6):e0268878. |
| Australia  n = 100 | Survey (telecommunication interaction) for community pharmacists’ appropriateness on giving clinical advice and anti-doping advice using a simulated-patient study design of a salbutamol user | A gap was identified regarding advising/counselling athletes, indicating the need for additional education in sport-related pharmacy. | Greenbaum, D.H., et al. (2023) International Journal of Pharmacy Practice. 31(3):290-7. |
